# Outpatient hydroxychloroquine prescribing at a large academic health system during the COVID-19 pandemic

**DOI:** 10.1017/ice.2020.243

**Published:** 2020-05-15

**Authors:** David R. Ha, Jamie Kuo, Phyo S. Aung, Tiffany Chang, Lana G. Witt, William Alegria, Amy Chang, Lina Meng, Stanley P. Deresinski

**Affiliations:** 1 Stanford Health Care, Stanford, California; 2 Medicine and Infectious Diseases, Stanford Medicine, Stanford, California

*To the Editor*—The coronavirus disease 2019 (COVID-19) pandemic has attracted widespread attention to experimental treatments, including the antirheumatic drug hydroxychloroquine, raising concerns about its supply for patients already taking the drug for non–COVID-19 indications.^1^ Currently, multiple manufacturers have reported shortages of hydroxychloroquine.^2^ We report an exploratory analysis of hydroxychloroquine prescribing in outpatient and urgent care clinics of a large academic health system in northern California.

## Methods

We conducted a retrospective, observational study of electronically documented outpatient hydroxychloroquine prescriptions originating from Stanford Medicine clinics between April 1, 2019, and March 31, 2020. Duplicate prescriptions for the same patient within a given month and those originating from emergency departments were excluded because data were unavailable. Prescriptions documented between March 24 and 31, 2020 were further assessed by chart review to determine indication for use.

## Results

**Fig. 1.**
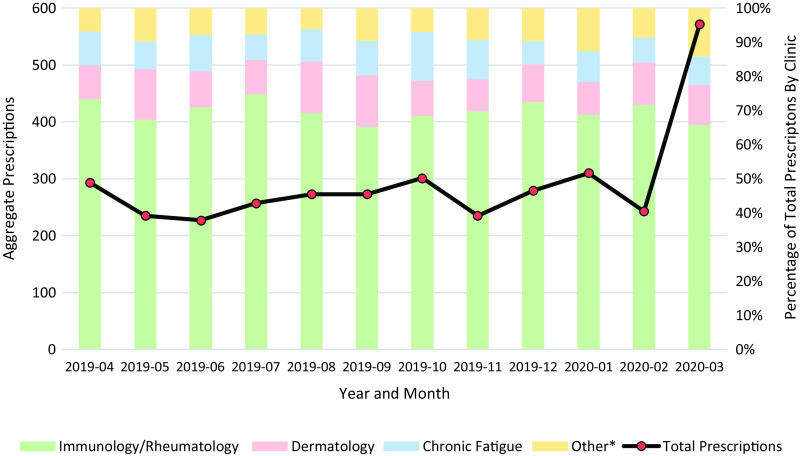
Hydroxychloroquine Prescriptions.

In total, 3,497 hydroxychloroquine prescriptions were included in this analysis. Over the 1-year study period, the highest prescribing clinics were the immunology–rheumatology (69%) and dermatology clinics (12%). Among all clinics, 571 hydroxychloroquine prescriptions were documented in March 2020, compared with a monthly mean of 266 (SD, 29) prescriptions in the preceding 11-month period. The distribution of prescriptions by clinic in March 2020 was similar to that in prior months (Fig. 1).

* Includes clinics whose prescription volume was less than 5% of all clinics. Includes the following clinics: Allergy, Blood and Marrow Transplant, Cardiovascular, Chest, Colorectal, Digestive Health, Endocrinology, Family Medicine, Heart Transplant, Hematology, Hepatology, Hospitalist, Infectious Diseases, Internal Medicine, Interventional Radiology, Kidney Transplant, Liver Transplant, Neurology, Nephrology, Oncology, Orthopedics, Otolaryngology, Pain, Pulmonology, Primary Care, Senior Care, and Urgent Care.

Among the 187 prescriptions documented between March 24 and 31, 2020, 169 (90%) were indicated as a continuation of ongoing therapy and 104 (56%) were written for a 90-day supply. Only 2 prescriptions were noted as being indicated for COVID-19 treatment.

## Discussion

We observed a >2-fold increase in electronically documented hydroxychloroquine prescriptions in March 2020 compared with the preceding 11-month period, despite only 2 of 187 prescriptions being used for COVID-19 from March 24 to 31, 2020. The immunology–rheumatology and dermatology clinics were the highest prescribers of hydroxychloroquine in our cohort, which appeared to be relatively constant in March 2020 compared with the preceding 11-month period. Most prescriptions documented between March 24 and 31, 2020, were for continuation of ongoing therapy in these clinics, primarily with 90-day supplies, consistent with ongoing therapy. Chart review revealed that some prescriptions were prompted by patient request due to concerns for drug shortage, lack of availability at pharmacies, and desire for alternate delivery modalities (eg, mail order).

Although our findings may reflect a desire to ensure continued supply for patients with chronic conditions, a trend in prescribing larger quantities may also negatively impact local drug supply. The CDC guidance has recommended that patients request larger prescription drug quantities to minimize pharmacy visits.^3^ However, the American College of Rheumatology has suggested limiting outpatient prescription refills of hydroxychloroquine to a 30-day supply as a potential mitigation strategy for any supply disruptions in select circumstances.^4^

Our analysis was observational in nature, and further interpretation is limited by several factors. Our data do not include non–electronically documented prescriptions (eg, phone call or hand written) or whether documented prescriptions were filled, and the data were not adjusted for patient or encounter volume. Indications of use, dosage, and other prescribing details were not assessed across the entire study period but were limited to a discrete period rather than a random sampling, which may have biased findings. Finally, these results are unique to practice paradigms of a single health system and are subject to regional epidemiology of COVID-19.

Understanding prescribing trends and impact on local drug supply during the COVID-19 pandemic may inform decisions by health systems, public health, insurers, and other governmental and healthcare organizations implementing strategies that promote appropriate utilization. As supported by pharmacy and medical associations, a better understanding of prescribing trends represents an important opportunity for interdisciplinary collaboration between pharmacists, physicians, and other healthcare professionals.^5^
